# Out-of-Pocket Expenses Related to Aging in Place for Older People Experiencing Frailty: A Scoping Review

**DOI:** 10.1093/geroni/igaa057.3233

**Published:** 2020-12-16

**Authors:** Elaine Moody, Rebecca Ganann, Marilyn Macdonald, Lori Weeks, Liz Orr, Keisha Jefferies, Jenny Ploeg, Ruth Martin Misener

**Affiliations:** 1 Dalhousie University, Halifax, Nova Scotia, Canada; 2 McMaster University, Hamilton, Ontario, Canada

## Abstract

Supporting older people to live in the community as they experience health and functional changes has become a priority for policy makers, health system leaders and community members, including many older people themselves. Aging-in-place has been promoted as a way to support the sustainability of health care systems and limit health care and societal costs. However, the expenses borne by individuals and caregivers to support older people to age-in-place when experiencing changes in health and functional ability are often not considered in health care literature and policy. We conducted a scoping review using Joanna Briggs Institute methodology to explore the out-of-pocket expenses for people with frailty living in the community. We included research and policy papers on community-dwelling people over 60 and experiencing frailty. Findings about financial out-of-pocket expenses were extracted. A total of 9669 sources were screened by two reviewers and 42 sources were included. The sources were from 17 countries, most from the US, and had various designs, including 14 qualitative designs, 15 cross sectional, 11 other quantitative and 2 policy discussions. The sources most often reported expenses related to home care (16), medication (12), housekeeping (10), transportation (8), and medical equipment (6). Gaps in the body of literature include lack of a consistent measure of out-of-pocket expenses and cost considerations of co-housing programs. The context—including policy, community and personal—was particularly important to the experience of out-of-pocket expenses for people with frailty, and further research is needed to expand on this knowledge.

